# Neural Dynamics Associated with Biological Variation in Normal Human Brain Regions

**DOI:** 10.3390/e26100828

**Published:** 2024-09-29

**Authors:** Natalí Guisande, Osvaldo A. Rosso, Fernando Montani

**Affiliations:** 1 Instituto de Física de La Plata (IFLP), CONICET-UNLP, La Plata B1900, Buenos Aires, Argentina; guisande.natali@fisica.unlp.edu.ar (N.G.); oarosso@gmail.com (O.A.R.); 2 Instituto de Física, Universidade Federal de Alagoas (UFAL), BR 104 Norte km 97, Maceió 57072-970, Brazil

**Keywords:** human iEEG, biological sex differences, shannon entropy, statistical complexity, information theory, power spectral density (PSD), brain dynamics, MNI open iEEG atlas, normal brain regions

## Abstract

The processes involved in encoding and decoding signals in the human brain are a continually studied topic, as neuronal information flow involves complex nonlinear dynamics. This study examines awake human intracranial electroencephalography (iEEG) data from normal brain regions to explore how biological sex influences these dynamics. The iEEG data were analyzed using permutation entropy and statistical complexity in the time domain and power spectrum calculations in the frequency domain. The Bandt and Pompe method was used to assess time series causality by associating probability distributions based on ordinal patterns with the signals. Due to the invasive nature of data acquisition, the study encountered limitations such as small sample sizes and potential sources of error. Nevertheless, the high spatial resolution of iEEG allows detailed analysis and comparison of specific brain regions. The results reveal differences between sexes in brain regions, observed through power spectrum, entropy, and complexity analyses. Significant differences were found in the left supramarginal gyrus, posterior cingulate, supplementary motor cortex, middle temporal gyrus, and right superior temporal gyrus. This study emphasizes the importance of considering sex as a biological variable in brain dynamics research, which is essential for improving the diagnosis and treatment of neurological and psychiatric disorders.

## 1. Introduction

A dynamic system is characterized by its evolving state over time, which can be fully described by a set of state variables at any given moment. These models allow for the analysis and prediction of system behavior under different inputs or conditions. Studying and understanding dynamic systems provides valuable insights into their behavior, allowing for predictions and even the control or optimization of their performance in various applications.

Dynamical systems can exhibit different dynamic properties, such as stability, periodicity, or chaos. Chaotic systems exhibit sensitivity to initial conditions, known as the butterfly effect, where small changes in initial conditions can lead to significant differences in the system’s behavior over time [1]. This is a fundamental characteristic shared by various physical systems, including neural networks, with the factors contributing to this sensitivity yet to be fully understood.

The dynamics of the brain show complex nonlinear characteristics, and their underlying mechanisms are still not well understood. While concrete evidence of chaos in cerebral dynamics, from a mathematical standpoint, has primarily been observed at the level of axons, individual cells, and paired cells, findings suggest that brain signals may exhibit chaotic patterns across all levels of their hierarchy [2,3,4].

Neural information processing is a challenging topic that requires an understanding of the intricate mechanisms underlying neuronal activity. One key aspect of this process is the nonlinear nature of neuronal dynamics, leading to complex phenomena such as chaos, synchronization, and bifurcations. These phenomena have significant implications for neural coding and computation. Analyzing the dynamics of nonlinear systems is not a straightforward task, especially when there are no available differential equations to model the system under study, as is the case with the brain.

One method of capturing the brain’s activity over time is through temporal recordings of its electrical activity via intercellular local field potentials (LFPs). These recordings reflect the extracellular electrical activity of nearby neurons and are obtained using deep electrodes inserted into the brain, resulting in intracranial electroencephalography (iEEG) signals, which are temporal voltage series.

The application of information theory to analyze iEEG signals enhances the understanding of brain activity by providing quantitative and objective measures. It enables the extraction of temporal characteristics and quantification of the information contained in the brain signals. Furthermore, information theory allows investigation of the complexity and organization of brain signals, revealing underlying patterns and organizational structures.

Utilizing ordinal patterns to compute measures based on permutation entropy enables the extraction of causal characteristics from signals and the assessment of nonlinear dynamics within these systems. The approach to computing the probability distribution function (PDF) from ordinal patterns, as proposed by Bandt and Pompe (BP), is widely embraced for electroencephalography (EEG) analysis, including iEEG, and has demonstrated superior results compared to conventional analyses [5].

Extensive studies have been conducted on non-intracranial EEG signals, such as the analysis of time series in epilepsy EEG [6,7], the distinction between brain death and coma [8], and the study of states of consciousness with the use of anesthetics [9], sleep stages [5,10,11,12], and signal discrimination [13,14,15]. In iEEG, this method has been applied to epilepsy for seizure prediction [16], detecting the epileptogenic focus [17], identifying preictal markers [18], extracting different characteristics for identifying the epileptogenic focus [17], and differentiating epileptic signals in an unsupervised manner [19,20,21]. These findings underscore the potential of permutation analysis in delving into diverse facets of EEG signal processing and analysis, empowered researchers to investigate the causal characteristics of signals, and evaluate the nonlinear dynamics within neural systems.

A growing body of research indicates sex-related differences in neural activity recorded in human EEG data [22,23,24,25,26,27,28,29,30,31,32,33,34,35,36,37,38,39,40,41,42,43,44]. Quantifiers derived from information theory, including markers based on the entropy of EEG background activity [45], have shown potential for identifying these sex differences. Several approaches have been proposed to exploit this potential, such as estimating differences in brain status through entropy measurements [46] and using permutation entropy to extract features for gender identification in emotional-based EEG datasets [47] and non-stationary EEG signals [48].

Applying these analyses to iEEG facilitates a deeper understanding of how men and women encode information during cognitive processes. A recent development involves creating a database to compile iEEG data from normal brain regions [49,50,51]. Given the impracticality of screening asymptomatic patients using invasive and costly methods, this database, comprising epileptic patients with identified epileptogenic foci and distinct healthy (non-affected) regions, currently provides the closest approximation for studying signals from normal areas.

iEEG signals, which record the brain’s electrical activity, are obtained through electrodes implanted in the cranial area or brain tissue. These signals offer superior spatial resolution and less tissue attenuation compared to EEGs, facilitating the precise localization of specific brain regions. They reflect the activity of ensembles or populations of neurons responsible for encoding sensory, motor, or cognitive information. Adopting the perspective of neural population [52] allows for investigating how the brain integrates and combines the activity of multiple neurons or brain regions to generate a coherent and efficient representation of internal or external stimuli.

This study aims to investigate how biological sex differences influence the way the brain encodes information through iEEG signals and to explore their impact on neuronal encoding dynamics. To achieve this, the study employs information theory quantifiers based on ordinal patterns that preserve causal coding features, using the Bandt and Pompe method. Complete signals are analyzed using the complexity-entropy causality plane and power spectral density. In each region, the mean, median, and Mann–Whitney U test were applied to entropy and complexity to compare between sexes. The analysis is based on iEEG signals sourced from healthy sectors of the human brain sourced from the Open iEEG Atlas [49,50,51].

The objective is to enhance the understanding of sex-based differences in brain activity through a comprehensive exploration of local field potentials. This involves the segregation of signals by sex, region, and hemisphere, followed by their analysis and the overlaying of results for graphical comparisons and statistical tests. Ultimately, the purpose of this study is to broaden the foundation for future research in neuroscience, thereby advancing comprehension of the observed diversity in the human brain and the influence of biological sex on cerebral dynamics.

## 2. Methods

### 2.1. Data Source and Preprocessing Methods

The data for this study were obtained from the MNI Open iEEG Atlas [49,50,51] database, which features recordings of intracranial activity in normal brain regions during various states, including quiet wakefulness with eyes closed, non-REM sleep (N2 and N3 stages), and REM sleep.

The signals were recorded from 1772 channels across 106 patients with focal epilepsy, using only those located in gray matter and considered “normal” (i.e., distant from epileptic regions). Various types of intracerebral electrodes were used, including Dixi, homemade MNI, AdTech electrodes, and AdTech subdural strips and grids.

The dataset includes patient information, such as sex, channel type, hemisphere, channel name, channel position, and channel region. Artifacts were detected and removed from the signals, and all signals were resampled at 200 samples per second to ensure consistency. Power-line interference was minimized using an adaptive filter. All channels were zero-padded to a length of 68 s (13,600 samples) to maintain uniform length regardless of the number of segments.

The electrodes were placed in a common stereotactic space to facilitate patient activity comparison and accumulation of results from multiple subjects. To obtain further information about the database and acquisition methods, the reader can refer to the following references [49,50,51].

### 2.2. Clustering of iEEG Data

The signals were separated by hemisphere, region, and sex, so that each region was analyzed in both hemispheres, with female and male analyzed separately. Only eyes-closed wakefulness data were analyzed in this study because there were no skipped channels or interruptions to consider when analyzing causality.

To compare male and female behavior within the same region, a condition was imposed requiring at least five patients of each sex. This was achieved by analyzing the signals without distinguishing between the electrodes used, as otherwise, fewer than two regions per hemisphere had more than five patients of each sex. All cases where this minimum statistic was not met were excluded from the analysis.

**Figure 1:**
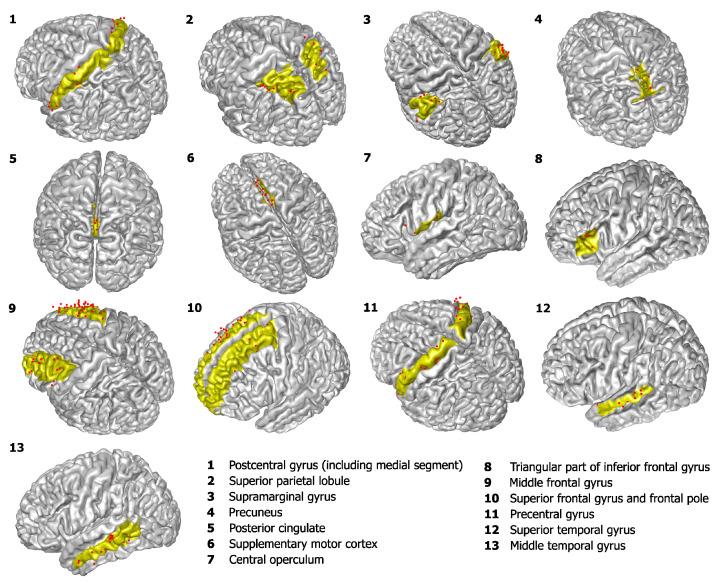
**Schema of the analyzed region locations along with the corresponding electrodes used.** The yellow area represents the region of interest, and the red dots indicate the positions of the electrodes.

Figure 1 provides a schematic representation of the brain, highlighting the localized regions that surpass the statistical restriction, as well as the corresponding electrodes used for channel analysis. The graphics were created based on the nodes of the surfaces and the electrode positions provided by the atlas [49,50,51].

### 2.3. Exploring iEEG Dynamics with Information Theory Tools

The study of neural systems and their dynamics is crucial for research in neuroscience. Understanding patterns and structures of neural activity can provide insights into brain function and potential mechanisms underlying neurological disorders. Information theory provides tools for analyzing neuronal activity by associating a probability distribution function (PDF) with the time series (TS) of brain signals. The Bandt and Pompe (BP) method offers a robust approach for processing nonlinear and noisy data while accounting for causality. This article uses the BP method to extract causal features from the signal and explore brain dynamics in both males and females. The iEEG TS is analyzed to quantify the information contained in observed neural activity by calculating Shannon entropy and statistical complexity. The following section discusses the information theory tools employed to analyze iEEG data, with a focus on the BP methodology used to obtain a probability distribution function for each signal while considering causality. Entropy and statistical complexity are then calculated from these distributions.

#### 2.3.1. Examining Ordinal Structure and Quantifying Information Using the Bandt-Pompe Approach

Quantifying the information contained in observed neural activity is essential for analyzing neural systems. This can be achieved by computing the Shannon entropy and statistical complexity of a TS associated with a PDF [53]. However, directly calculating a histogram from the signal may lead to information loss concerning the temporal causality of the underlying dynamical system. Fortunately, the BP approach offers a robust alternative for analyzing nonlinear and noisy data, including neural activity [54]. This method utilizes the ordinal structure of the TS to derive a PDF of ordinal patterns, which captures the underlying structure of the TS without assuming any specific model. By comparing the relative values of the points in the TS rather than the amplitudes, this methodology constructs the PDF associated with the TS using two parameters: embedding dimension and embedding delay.

In this study, the BP technique is used to estimate the PDF of an iEEG TS, $\chi \left(t\right)\;=\;\left\{x_{t};\;t=1,\cdots ,M\right\}$, comprising *M* observations, as described in [54,55,56,57]. The BP approach involves transforming the TS into symbolic sequences and identifying ordinal structures by considering all possible permutations of the series values within fixed-size windows. The resulting PDF of ordinal patterns is obtained by calculating histograms of patterns in the signal that reflect causality.

The TS is partitioned into n=M−(D−1)τ overlapping segments, each with a predetermined embedding dimension (*D*) and embedding delay (τ). Each segment is represented by a *D*-dimensional vector of values, from which the permutation of index numbers is determined. Every possible permutation of order *D* (with D! permutations) is considered, and its relative frequency is computed to generate a histogram of ordinal patterns. This distribution reflects the structure of the TS and can be used to compute the Shannon entropy and statistical complexity of the observed neural activity.

Finally, to calculate the PDF associated with the TS, the following expression can be used:

$$p_{j}\left(\Pi_{j}\right)=\frac{\mathrm{number}\;\mathrm{of}\;\mathrm{partitions}\;\mathrm{of}\;\mathrm{type}\;\Pi_{j}\;\mathrm{in}\;\pi_{i}}{n}\;,$$

where $p_{j}\left(\Pi_{j}\right)$ represents the relative frequency of the *j*-th ordinal pattern $\Pi_{j}$, and $\pi_{i}$ denotes the sequence of all ordinal patterns for all partitions. Note that the estimated PDF *P* is discrete since it is derived from a histogram. Additionally, the TS analyzed, χ(t), should be much longer than the number of possible ordinal patterns (M>>D! where M is the number of samples in the TS) to ensure good statistical results when applying this technique. Readers interested in further information and examples of the BP methodology can refer to the following references: [53,54,55,56,57,58].

#### 2.3.2. Quantifying Information-Theoretic Measures: Entropy and Complexity Calculations

Quantifying information-theoretic measures is crucial for understanding the dynamics of a system represented by its TS. Although permutation Shannon entropy effectively measures randomness in a system, it may not fully capture ordinal structures. Therefore, measures of statistical or structural complexity are needed for a more comprehensive characterization of the system’s dynamics [59].

In this study, the BP methodology is used to estimate the underlying PDF of a TS, χ(t), and two information-theoretic quantifiers are calculated using the ordinal patterns method to detect and quantify ordinal structures in the TS. These quantifiers enable a comparison of the dynamics of physiological signals in the human brain between biological sexes.

Shannon entropy measures randomness in a TS and is calculated using its PDF. In the discrete case, the Shannon entropy of a TS with a corresponding PDF $P\equiv {p_{j}}_{\left\{j=1,\cdots ,N\right\}}$ is given by the equation $S\left[P\right]\;=\;-\sum_{j=1}^{N}p_{j}\log_{2}\left(p_{j}\right)$, where *N* is the number of possible states and $p_{j}$ is the probability of each state. However, large changes in *P* over a small range have minimal impact on the Shannon entropy, as it is a “global” measure.

To more accurately characterize the system’s dynamics, measures of statistical or structural complexity are needed. Statistical complexity, developed by López-Ruiz et al. [60], captures subtle differences in the dynamics of the systems under study. This study uses the version of the metric developed by Martin-Rosso-Plastino, known as MPR complexity [61,62]. From this point on, the term “complexity” refers specifically to this metric.

Statistical complexity is computed using $C=Q_{J}\left[P,P_{e}\right]\cdot H\left[P\right]$. The normalized Shannon entropy *H*, is defined as $H\left[P\right]=\frac{S[P]}{Smax}$, where $S_{max}\;=\;S\left[P_{e}\right]\;=\;\log_{2}N$. The disequilibrium ($Q_{J}=Q_{0}J$) is a function of the Jensen–Shannon divergence, given as $J\left[P,P_{e}\right]\;=\;H\left[\frac{P+P_{e}}{2}\right]-\frac{H[P]}{2}-\frac{H[P_{e}]}{2}$, and a normalization constant $Q_{0}$. Here, $P_{e}$ refers to the uniform distribution. To obtain the statistical complexity, $Q_{J}$ and *H* are multiplied together.

Statistical complexity and entropy were calculated for channels within the same region, hemisphere, and biological sex. To traverse the TS, non-overlapping time windows smaller than the original signals were utilized. A window size of 15 s was chosen to balance the need for maximum causality (M>>D!) with minimal loss of information in neural encoding [63]. This choice corresponded to 3000 samples, given the signals’ sampling frequency of 200 Hz.

The BP methodology was applied to each window to obtain a PDF that accounts for the causality of the signal. For this analysis, D=6 and τ=1 were used. Complexity and entropy were then calculated from these PDFs. The values were accumulated for patients meeting the specified conditions, and the results were plotted for both males and females. In each region, the mean, median, and Mann–Whitney *U* test were utilized to quantify any differences found between the results for males and females.

To further validate the condition M>>D!, the same analysis was conducted with an embedding dimension of D=5, using the same time window and τ=1.

### 2.4. Investigating Frequency Bands in iEEG Signals: Power Spectral Density Analysis Using the Welch Method

In the study of neuroscientific phenomena, power spectral density (PSD) is frequently utilized as a measure of the distribution of power in a signal across different frequencies. This method is commonly applied to electroencephalography (EEG) and intracranial EEG (iEEG) signals to gain insight into underlying neural processes. PSD analysis can be employed in iEEG to examine the frequency content of LFPs and identify neural processes associated with specific frequency bands.

The Welch method [64] is a widely used approach for estimating PSD. It involves dividing the signal into overlapping segments, applying a window function to each segment to reduce spectral leakage, computing a periodogram for each segment, and then averaging the periodograms to estimate the PSD. A periodogram is a mathematical tool that breaks down a signal into its component frequencies, helping to identify which frequencies are present and their strengths. The Fourier transform is commonly employed to compute periodograms, decomposing signals into sine and cosine waves of varying amplitude and frequency.

Welch’s approach is particularly useful for analyzing non-stationary signals as it allows for the estimation of the PSD at multiple time points. To estimate the PSD of iEEG signals using the Welch method, the iEEG signal is first divided into overlapping segments of equal length. A window function, such as the Hamming window, is then applied to each segment to reduce spectral leakage, and the periodogram of each segment is computed using the fast Fourier transform (FFT) method. The periodograms are then averaged across the segments to obtain an estimate of the PSD. For further information on these methods and their implementation, refer to the literature cited in Refs. [65,66].

In this work, Welch’s method was used to estimate the PSD of the iEEG. Specifically, the magnitude of the discrete-time Fourier transform of 59 overlapping blocks of 2 s duration and 1 s step, weighted by a Hamming window, was averaged to obtain the spectral density in each channel. The resulting spectral density in each channel was then normalized to a total power, ensuring independence from the amplitude of the iEEG signal. The selection of these parameters was based on the work by the creators of the MNI Open iEEG Atlas (see Refs. [49,50,51]).

## 3. Results

This section presents the results of Shannon entropy and statistical complexity across different brain regions in the left and right hemispheres of both male and female individuals, using embedding dimensions of D=6 and D=5, with a time window of 15 s and a delay of τ=1. Additionally, power spectra and the complexity-entropy plane are shown for those regions where significant differences between sexes were identified using the Mann–Whitney *U* test.

The first step involved examining the age distribution of the patients to account for any potential age-related differences in the results. Table 1 displays the number of patients (n), mean age, and standard deviation for each region. Within each region, the age ranges for males and females were similar and fell within the standard deviation, which supported the assumption that no significant disturbances in the calculated quantifiers arose from age differences.

Differences in Shannon entropy and statistical complexity across different brain regions in the left and right hemispheres of male and female individuals are presented in Table 2. The analysis was conducted using the embedding dimension D=6. Only regions with a minimum of five female and five male patients were included in the analysis. For each region, the mean and median values of Shannon entropy and statistical complexity were calculated separately for males and females. The statistical significance of the differences between male and female values was assessed using the Mann–Whitney *U* test, with the results presented as *p*-values and *h*-index. Significant differences in both Shannon entropy and statistical complexity between males and females were observed in certain brain regions, as highlighted in the table. Conversely, the remaining regions showed no significant differences between sexes for either measure.

The following figures display the PSD and complexity-entropy causality plane for various brain regions in the left and right hemispheres. Each figure shows results from both male and female patients, normalized to the total power in each channel. The format is consistent across all figures: (A) and (B) present the median PSD for female and male patients respectively (where the shaded area represents the interquartile range, IQR), (C) shows the median PSD for both sexes, and (D) illustrates the complexity-entropy causality plane for each sex using D=6. Each scatter plot includes a boxplot for each sex, showing the median, IQR, outliers, and the notch, which indicates the 95% confidence interval of the median.

**Figure 2:**
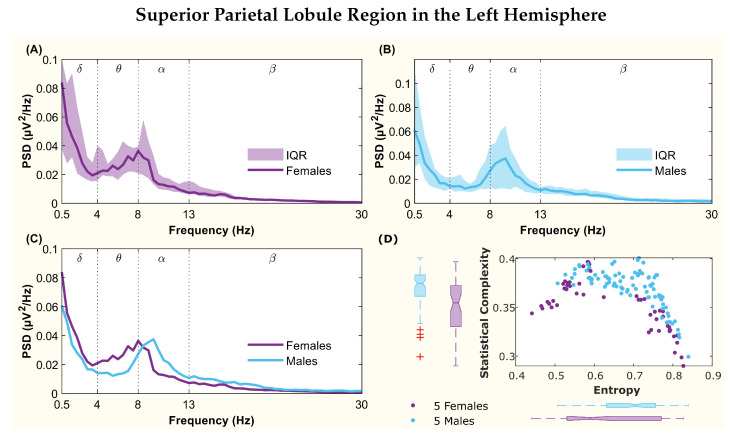
**Power spectral density (PSD) and complexity-entropy causality plane of the superior parietal lobule region in the left hemisphere.** (**A**,**B**) PSD of female and male patients, respectively, with the solid line representing the median and the shaded area representing the interquartile range (IQR). (**C**) Median PSD of females and males. (**D**) Complexity-entropy causality plane using an embedding dimension D=6, a time delay τ=1, and a temporal window of 15 s for females and males. The boxplot shows the median, IQR, outliers, and the notch, which corresponds to the 95% confidence interval of the median.

**Figure 3:**
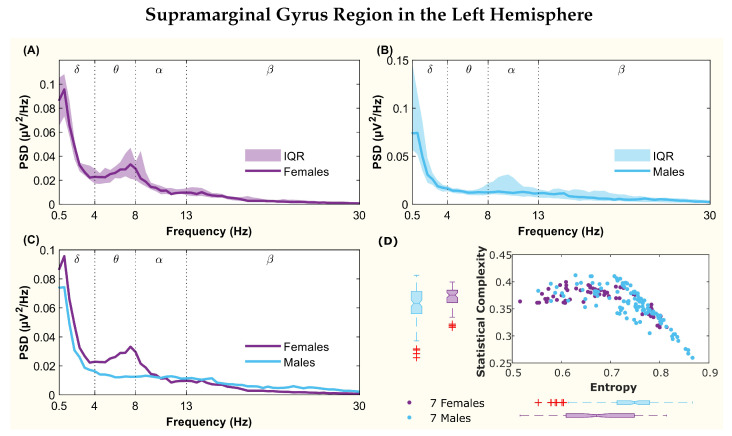
**Power spectral density (PSD) and complexity-entropy causality plane of the supramarginal gyrus region in the left hemisphere.** (**A**,**B**) PSD of female and male patients, respectively, with the solid line representing the median and the shaded area representing the interquartile range (IQR). (**C**) Median PSD of females and males. (**D**) Complexity-entropy causality plane using an embedding dimension D=6, a time delay τ=1, and a temporal window of 15 s for females and males. The boxplot shows the median, IQR, outliers, and the notch, which corresponds to the 95% confidence interval of the median.

**Figure 4:**
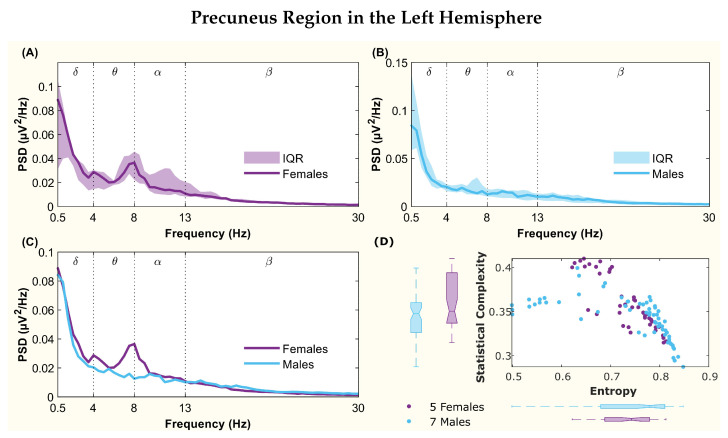
**Power spectral density (PSD) and complexity-entropy causality plane of the precuneus region in the left hemisphere.** (**A**,**B**) PSD of female and male patients, respectively, with the solid line representing the median and the shaded area representing the interquartile range (IQR). (**C**) Median PSD of females and males. (**D**) Complexity-entropy causality plane using an embedding dimension D=6, a time delay τ=1, and a temporal window of 15 s for females and males. The boxplot shows the median, IQR, outliers, and the notch, which corresponds to the 95% confidence interval of the median.

**Figure 5:**
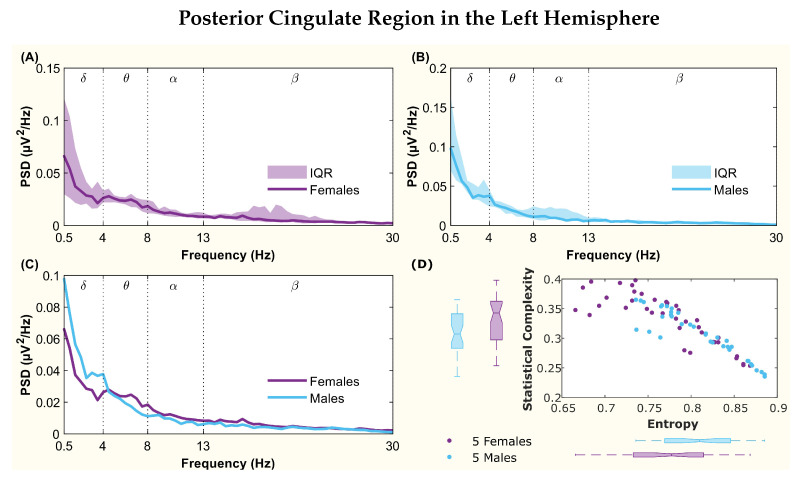
**Power spectral density (PSD) and complexity-entropy causality plane of the posterior cingulate region in the left hemisphere.** (**A**,**B**) PSD of female and male patients, respectively, with the solid line representing the median and the shaded area representing the interquartile range (IQR). (**C**) Median PSD of females and males. (**D**) Complexity-entropy causality plane using an embedding dimension D=6, a time delay τ=1, and a temporal window of 15 s for females and males. The boxplot shows the median, IQR, outliers, and the notch, which corresponds to the 95% confidence interval of the median.

**Figure 6:**
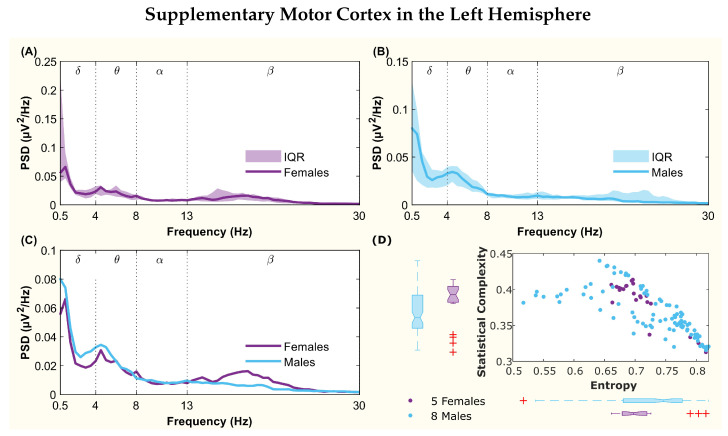
**Power spectral density (PSD) and complexity-entropy causality plane of the supplementary motor cortex in the left hemisphere.** (**A**,**B**) PSD of female and male patients, respectively, with the solid line representing the median and the shaded area representing the interquartile range (IQR). (**C**) Median PSD of females and males. (**D**) Complexity-entropy causality plane using an embedding dimension D=6, a time delay τ=1, and a temporal window of 15 s for females and males. The boxplot shows the median, IQR, outliers, and the notch, which corresponds to the 95% confidence interval of the median.

**Figure 7:**
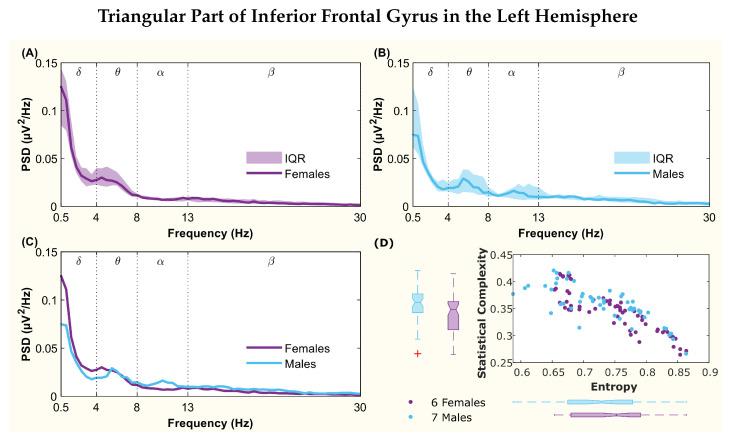
**Power spectral density (PSD) and complexity-entropy causality plane of the triangular part of the inferior frontal gyrus in the left hemisphere.** (**A**,**B**) PSD of female and male patients, respectively, with the solid line representing the median and the shaded area representing the interquartile range (IQR). (**C**) Median PSD of females and males. (**D**) Complexity-entropy causality plane using an embedding dimension D=6, a time delay τ=1, and a temporal window of 15 s for females and males. The boxplot shows the median, IQR, outliers, and the notch, which corresponds to the 95% confidence interval of the median.

**Figure 8:**
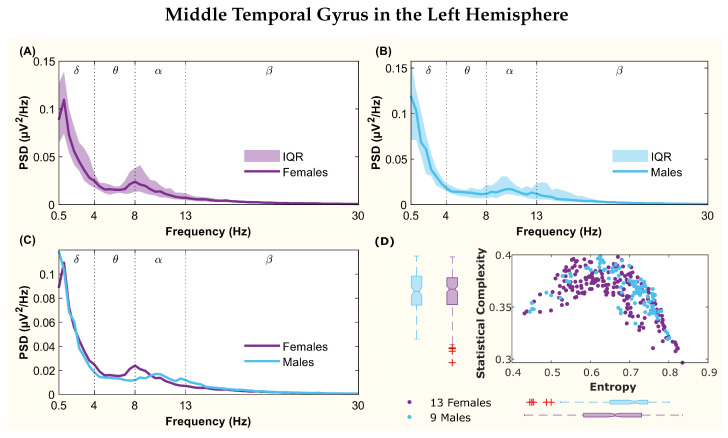
**Power spectral density (PSD) and complexity-entropy causality plane of the middle temporal gyrus in the left hemisphere.** (**A**,**B**) PSD of female and male patients, respectively, with the solid line representing the median and the shaded area representing the interquartile range (IQR). (**C**) Median PSD of females and males. (**D**) Complexity-entropy causality plane using an embedding dimension D=6, a time delay τ=1, and a temporal window of 15 s for females and males. The boxplot shows the median, IQR, outliers, and the notch, which corresponds to the 95% confidence interval of the median.

**Figure 9:**
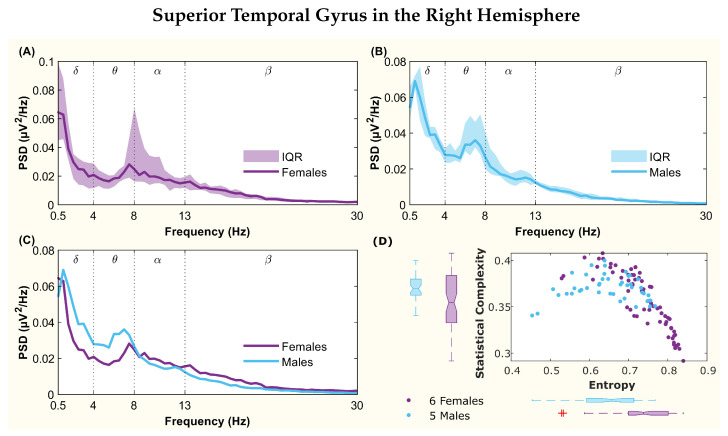
**Power spectral density (PSD) and complexity-entropy causality plane of the superior temporal gyrus in the right hemisphere.** (**A**,**B**) PSD of female and male patients, respectively, with the solid line representing the median and the shaded area representing the interquartile range (IQR). (**C**) Median PSD of females and males. (**D**) Complexity-entropy causality plane using an embedding dimension D=6, a time delay τ=1, and a temporal window of 15 s for females and males. The boxplot shows the median, IQR, outliers, and the notch, which corresponds to the 95% confidence interval of the median.

The figures correspond to the brain regions highlighted in Table 2, which showed significant differences between males and females based on the statistical test with D=6. These regions include the superior parietal lobule (Figure 2), supramarginal gyrus (Figure 3), precuneus (Figure 4), posterior cingulate (Figure 5), supplementary motor cortex (Figure 6), the triangular part of the inferior frontal gyrus (Figure 7), and the middle temporal gyrus in the left hemisphere (Figure 8), as well as the superior temporal gyrus in the right hemisphere (Figure 9).

In Table 2, using D=6, it can be seen that differences between the entropy and statistical complexity results for both biological sexes were observed in half of the analyzed regions, as quantified by a Mann–Whitney test. The normalized spectral analysis of the signals also revealed differences between the median results for both sexes. The figures provide a qualitative analysis, showing not only the medians but also the dispersion of the data through interquartile ranges (IQRs).

In the left hemisphere, seven regions with significant differences were identified for D=6. In the superior parietal lobule, the median complexity and entropy values showed disjoint notches in the boxplots, and the statistical test indicated that the entropy values come from different distributions (Figure 2D). Figure 2C shows that the PSD had a peak for both men and women. For women, this peak was located at the boundary of the θ−α bands, while for men, it was shifted towards the α band.

The supramarginal gyrus region exhibited differences in the PSD spectra. A peak was observed in the spectrum for women at the end of the θ band, while the male spectrum lacked peaks in both the θ and γ bands (Figure 3C). The complexity-entropy plane also showed differences, with notched medians indicating distinct distributions for men and women (Figure 3D), as confirmed by statistical testing.

Similar patterns were observed in the precuneus region. The female PSD displayed a peak between the θ−α bands, whereas no peaks were seen in the male PSD within these regions (Figure 4C). Although the notches in the medians had some overlap (Figure 4D), the statistical test revealed differences in entropy values between men and women.

In the posterior cingulate region (Figure 5), both spectra showed a small peak in the boundary between the δ and θ bands, with the male peak shifted to slightly lower frequencies (Figure 5C). Regarding complexity and entropy (Figure 5D), while some overlap was observed between the notches in entropy, the values for complexity were well separated. Statistical tests indicated that the distributions for both quantifiers were different.

The supplementary motor cortex presented a peak at the beginning of the θ band in both spectra. However, as frequencies increased, differences emerged: the female spectrum showed a peak in the β band, which was absent in the male spectrum (Figure 6C). Although the medians for complexity and entropy were well differentiated with non-overlapping notches, statistical tests revealed significant differences only for entropy (Figure 6D).

In the triangular part of the inferior frontal gyrus region, both spectra showed a peak in the θ band, but the female peak was shifted toward lower frequencies. Additionally, the male PSD featured a second peak centered in the α band which was not present in the female spectrum (Figure 7). Despite overlapping notches in the medians for complexity and entropy, statistical tests indicated differences between sexes for entropy.

The middle temporal gyrus region showed differences between the θ and α bands in the PSD. A peak in the female spectrum occurred at the boundary between these bands, whereas the male spectrum displayed two peaks in the α band (Figure 8C). While entropy medians were well differentiated, complexity medians presented overlapping notches (Figure 8D). Statistical tests revealed significant differences in complexities, but not in entropies.

In the right hemisphere, among the four regions with acceptable statistics, only the superior temporal gyrus region displayed differences for D=6. Both spectra showed a peak, with the female peak located between the θ and α bands, and the male peak shifted to the left in the θ band with higher amplitude (Figure 9C). Non-overlapping notches for both entropy and complexity were observed (Figure 9D), but statistical tests revealed significant differences only for complexity.

To improve the statistics of BP and ensure that the length of the time series (M) was much greater than D!, an embedding dimension of D=5 was used with the same time window. Results for mean, median, and statistical tests of Shannon entropy and statistical complexity using D=5 are presented in Table 3.

With D=5, the regions of the supramarginal gyrus, posterior cingulate, supplementary motor cortex, and middle temporal gyrus in the left hemisphere showed differences between males and females. Shannon entropy differences were detected in the first three regions, while all four regions displayed differences in statistical complexity. In the right hemisphere, differences were observed in two regions: the superior temporal gyrus, which showed differences in both quantifiers, and the middle temporal gyrus, which showed differences in entropy.

In summary, significant differences were observed in both D=6 and D=5 analyses in the following regions: the supramarginal gyrus, the posterior cingulate, the supplementary motor cortex, and the middle temporal gyrus in the left hemisphere, and the superior temporal gyrus in the right hemisphere.

## 4. Discussions

In summary, the findings of this study indicate that biological sex-based differences in brain function can be detected through iEEG signal analysis. These variations are evident in multiple brain regions, affecting both spectral characteristics and measures of complexity and entropy.

Further research is needed to explore the underlying causes of these differences. The invasive nature of iEEG signal collection prevents its application to healthy subjects, resulting in a restricted amount of LFP data from normal brain regions. However, despite constraints on patient numbers per region, iEEG analysis offers unparalleled spatial resolution compared to EEG, enabling the capture of region-specific LFPs and allowing for comparisons across sexes. Additionally, signals collected from electrodes implanted directly in the brain minimize attenuation caused by the skull and other tissues, a common issue with EEG, leading to less interference. These advantages come with challenges, as the “healthy” regions analyzed originate from the brains of epilepsy patients. This not only limits the dataset size but may also introduce bias, as the signals are taken from regions deemed “normal”, rather than from truly “healthy” individuals.

To address potential factors influencing the results, a strategy was employed to adjust for age within the analyzed sample, despite its limited size. The dataset was subsampled to include five patients for each region, aiming to minimize age differences between males and females while reducing standard deviations. The results of this analysis are detailed in this Section for the interested reader. The tables for D=6 show that the number of regions exhibiting differences increased under this restriction (see Table 4 and Table 5) in both hemispheres. Additionally, Table 6 provides the results of a significance analysis using the Benjamini–Hochberg FDR correction for D=5 and D=6, applied to both the entire patient cohort and the subsets created to minimize differences and deviations. Reducing age differences in the dataset helps to minimize the impact of age-related variables or confounding factors that could introduce noise or skew the results. By reducing age variability between groups (i.e., males and females), the analysis can more directly focus on the effects of the variables of interest, reducing the influence of age as a potential source of noise or bias. This approach also increases sensitivity to variations attributable to biological sex, reinforcing the current findings.

Neuroscience research must increasingly acknowledge the vast diversity present in human brains. A key step in this direction is adopting a binary, proportional, and inclusive segmentation based on common biological classifications of male and female. To better understand brain dynamics, it is essential to reduce biases in sample selection and analyze the data both collectively and separately. Future research should also investigate whether these differences persist when considering perceived gender identity and hormonal backgrounds, as these factors can significantly influence brain structure and function. Incorporating these variables could provide a more comprehensive understanding of brain dynamics and help mitigate biases in neuroscience research.

This study found that both Shannon entropy and MPR statistical complexity can serve as sensitive biomarkers for biological sex in iEEG from awake patients. Recently, we proposed a framework constructed with Renyi entropy and its generalized statistical complexity, where we detected sex-based differences related to scale-free phenomena in non-REM sleep stages N1 and N2, as well as REM sleep [67].

These findings underscore the necessity of addressing biases in experimental design. Including both males and females in the study population is crucial for ensuring that results are representative and not skewed by gender imbalances. Differences in average brain dynamics between sexes highlight the importance of inclusivity and equality in research. Despite advancements in gender equality, preclinical neuroscience often inadvertently excludes women, leading to biased data and conclusions. Thus, considering biological sex and gender differences is essential for obtaining a comprehensive understanding of brain function. Incorporating these variables into future studies could enhance our understanding of brain dynamics and contribute to reducing biases in neuroscience research.

## 5. Conclusions

This study examined biological sex differences in statistical complexity MPR, Shannon entropy, and power spectral density (PSD) across various regions of the brain. Significant variations in Shannon entropy and statistical complexity were detected between males and females in certain brain areas, with these differences persisting across multiple analyses. The regions where these distinctions were observed, both in power spectra analyses and when computing entropy and complexity with embedding dimensions D=5 and D=6, include the supramarginal gyrus, posterior cingulate, supplementary motor cortex, and middle temporal gyrus in the left hemisphere, and in the right hemisphere, the superior temporal gyrus.

Overall, this study supports the notion that biological sex-based distinctions exist in brain function. Furthermore, it suggests that statistical complexity, Shannon entropy, and PSD can serve as sensitive biomarkers for identifying these variations. Differences related to biological sex and scale-free dynamics have also been found in this atlas when analyzing non-REM stages N2 and N3, as well as REM sleep, in another study [67]. Moreover, due to limited data, this study did not consider the impact of gender identity on the outcomes, and the number of patients by area was limited. Future investigations will aim for a more comprehensive analysis, including filtering by frequency bands and exploring high-frequency oscillation behavior to compare neural activity between sexes.

This research underscores the importance of recognizing sex as a biological variable when designing and conducting studies in the field of neuroscience.

## Figures and Tables

**Table 1:** Mean ages (in years) of patients in each analyzed region, along with their respective standard deviation. ’n’ represents the number of patients.

**Left Hemisphere**						
	**Female**			**Male**		
**Region**	**n**	**Mean**	**std**	**n**	**Mean**	**std**
Superior parietal lobule	5	30	10	5	40	16
Supramarginal gyrus	7	27	9	7	40	12
Precuneus	5	26	9	7	30	12
Posterior cingulate	5	30	11	5	40	15
Supplementary motor cortex	5	29	7	8	37	9
Central operculum	5	24	6	8	35	9
Triangular part of inferior frontal gyrus	6	30	10	7	35	7
Middle frontal gyrus	10	29	8	13	36	6
Superior frontal gyrus and frontal pole	5	26	7	8	38	7
Precentral gyrus	6	25	6	12	30	11
Superior temporal gyrus	12	30	11	5	36	4
Middle temporal gyrus	13	30	10	9	35	7
**Right Hemisphere**						
	**Female**			**Male**		
**Region**	**n**	**mean**	**std**	**n**	**mean**	**std**
Postcentral gyrus (including medial segment)	7	20	10	7	30	11
Middle frontal gyrus	6	40	15	17	34	8
Superior temporal gyrus	6	30	12	5	30	10
Middle temporal gyrus	8	30	11	8	35	11

**Table 2:** Comparison of Shannon entropy and statistical complexity between males and females across different brain regions using D=6.

**Left Hemisphere**												
	**Shannon Entropy**						**Statistical Complexity**					
**Region**	**Female**		**Male**		**Test**		**Female**		**Male**		**Test**	
	**Mean**	**Median**	**Mean**	**Median**	***p*-Value**	**h**	**Mean**	**Median**	**Mean**	**Median**	***p*-Value**	**h**
**Superior parietal lobule**	0.35	0.35	0.37	0.37	$1\times 10^{-5}$	1	0.64	0.59	0.69	0.70	0.05	0
**Supramarginal gyrus**	0.37	0.38	0.36	0.36	0.02	1	0.68	0.67	0.74	0.75	$2\times 10^{-6}$	1
**Precuneus**	0.36	0.35	0.34	0.35	0.04	1	0.73	0.74	0.73	0.78	0.05	0
**Posterior cingulate**	0.33	0.34	0.31	0.31	0.01	1	0.77	0.78	0.81	0.81	0.01	1
**Supplementary motor cortex**	0.39	0.39	0.37	0.36	0.01	1	0.71	0.70	0.72	0.75	0.05	0
**Central operculum**	0.36	0.38	0.37	0.37	0.81	0	0.69	0.71	0.69	0.70	0.70	0
**Triangular part of inferior frontal gyrus**	0.34	0.35	0.36	0.36	0.03	1	0.75	0.75	0.73	0.73	0.19	0
**Middle frontal gyrus**	0.35	0.35	0.35	0.36	0.31	0	0.75	0.76	0.74	0.75	0.89	0
**Superior frontal gyrus and frontal pole**	0.36	0.36	0.36	0.37	0.92	0	0.72	0.71	0.70	0.72	0.50	0
**Precentral gyrus**	0.38	0.39	0.36	0.38	0.15	0	0.70	0.71	0.71	0.70	0.96	0
**Superior temporal gyrus**	0.36	0.36	0.36	0.36	0.64	0	0.66	0.68	0.65	0.66	0.43	0
**Middle temporal gyrus**	0.36	0.37	0.37	0.36	0.64	0	0.66	0.66	0.69	0.71	$3\times 10^{-3}$	1
**Right Hemisphere**												
	**Shannon Entropy**						**Statistical Complexity**					
**Region**	**Female**		**Male**		**Test**		**Female**		**Male**		**Test**	
	**mean**	**median**	**mean**	**median**	***p*-Value**	**h**	**mean**	**median**	**mean**	**median**	***p*-Value**	**h**
**Postcentral gyrus (including medial segment)**	0.37	0.37	0.38	0.38	0.07	0	0.66	0.65	0.68	0.68	0.29	0
**Middle frontal gyrus**	0.35	0.35	0.36	0.37	0.08	0	0.76	0.78	0.74	0.76	0.10	0
**Superior temporal gyrus**	0.36	0.35	0.37	0.37	0.06	0	0.74	0.74	0.65	0.66	$3\times 10^{-7}$	1
**Middle temporal gyrus**	0.34	0.35	0.35	0.35	0.12	0	0.73	0.76	0.71	0.73	0.06	0

**Table 3:** Comparison of Shannon entropy and statistical complexity between males and females across different brain regions using D=5.

**Left Hemisphere**												
	**Shannon Entropy**						**Statistical Complexity**					
**Region**	**Female**		**Male**		**Test**		**Female**		**Male**		**Test**	
	**Mean**	**Median**	**Mean**	**Median**	***p*-Value**	**h**	**Mean**	**Median**	**Mean**	**Median**	***p*-Value**	**h**
**Superior parietal lobule**	0.27	0.30	0.28	0.28	0.98	0	0.68	0.63	0.73	0.74	0.09	0
**Supramarginal gyrus**	0.28	0.29	0.26	0.26	0.001	1	0.71	0.71	0.77	0.78	2 $\times \;10^{-6}$	1
**Precuneus**	0.26	0.25	0.25	0.24	0.10	0	0.77	0.78	0.77	0.81	0.07	0
**Posterior cingulate**	0.23	0.24	0.20	0.21	0.01	1	0.81	0.81	0.84	0.84	0.01	1
**Supplementary motor cortex**	0.29	0.30	0.27	0.26	0.05	1	0.74	0.73	0.76	0.78	0.05	1
**Central operculum**	0.27	0.29	0.28	0.28	0.90	0	0.73	0.75	0.73	0.73	0.74	0
**Triangular part of inferior frontal gyrus**	0.25	0.25	0.26	0.26	0.05	0	0.78	0.79	0.76	0.77	0.15	0
**Middle frontal gyrus**	0.25	0.25	0.25	0.26	0.30	0	0.78	0.79	0.78	0.79	0.96	0
**Superior frontal gyrus and frontal pole**	0.27	0.26	0.27	0.27	0.58	0	0.76	0.75	0.74	0.76	0.52	0
**Precentral gyrus**	0.29	0.29	0.27	0.29	0.49	0	0.74	0.74	0.74	0.74	0.99	0
**Superior temporal gyrus**	0.28	0.29	0.28	0.28	0.79	0	0.69	0.72	0.69	0.70	0.47	0
**Middle temporal gyrus**	0.28	0.29	0.27	0.27	0.10	0	0.69	0.70	0.72	0.75	0.003	1
**Right Hemisphere**												
	**Shannon Entropy**						**Statistical Complexity**					
**Region**	**Female**		**Male**		**Test**		**Female**		**Male**		**Test**	
	**mean**	**median**	**mean**	**median**	***p*-Value**	**h**	**mean**	**median**	**mean**	**median**	***p*-Value**	**h**
**Postcentral gyrus (including medial segment)**	0.29	0.29	0.29	0.30	0.64	0	0.69	0.69	0.72	0.72	0.30	0
**Middle frontal gyrus**	0.25	0.25	0.26	0.26	0.07	0	0.80	0.81	0.77	0.79	0.10	0
**Superior temporal gyrus**	0.26	0.26	0.29	0.29	2 $\times \;10^{-4}$	1	0.77	0.77	0.69	0.70	4 $\times \;10^{-7}$	1
**Middle temporal gyrus**	0.24	0.24	0.26	0.26	0.05	1	0.77	0.79	0.75	0.76	0.05	0

**Table 4:** Mean ages (in years) of patients selected to minimize differences in mean values between males and females for each analyzed region, along with their respective standard deviations.

**Left Hemisphere**				
	**Female**		**Male**	
**Region**	**Mean**	**sd**	**Mean**	**sd**
Superior parietal lobule	29	10	41	16
Supramarginal gyrus	30	9	32	10
Precuneus	26	9	29	8
Posterior cingulate	28	11	38	15
Supplementary motor cortex	29	7	33	10
Central operculum	24	6	31	8
Triangular part of inferior frontal gyrus	33	10	33	7
Middle frontal gyrus	31	9	31	6
Superior frontal gyrus and frontal pole	26	7	34	6
Precentral gyrus	26	6	26	6
Superior temporal gyrus	36	13	36	4
Middle temporal gyrus	34	8	34	5
**Right Hemisphere**				
	**Female**		**Male**	
**Region**	**mean**	**sd**	**mean**	**sd**
Postcentral gyrus (including medial segment)	27	10	27	8
Middle frontal gyrus	37	16	37	8
Superior temporal gyrus	32	13	33	10
Middle temporal gyrus	36	9	36	13

**Table 5:** Comparison of Shannon entropy and statistical complexity between males and females across different brain regions, using D = 6 for the five patients of each sex selected to minimize differences in mean age values and standard deviations. h: null hypothesis; sd: standard deviation. Light gray shading indicates regions with sex differences in one quantifier, while bold gray shading represents regions where both quantifiers showed sex differences.

**Left Hemisphere**																
	**Shannon Entropy**								**Statistical Complexity**							
**Region**	**Female**			**Male**			**Test**		**Female**			**Male**			**Test**	
	**Mean**	**Median**	**sd**	**Mean**	**Median**	**sd**	***p*-Value**	**h**	**Mean**	**Median**	**sd**	**Mean**	**Median**	**sd**	***p*-Value**	**h**
**Superior parietal lobule**	0.64	0.59	0.13	0.69	0.705	0.081	0.05	0	0.352	0.355	0.026	0.370	0.375	0.020	1 $\times \;10^{-5}$	1
**Supramarginal gyrus**	0.675	0.657	0.090	0.76	0.758	0.051	3 $\times \;10^{-6}$	1	0.366	0.368	0.022	0.355	0.354	0.035	0.09	0
**Precuneus**	0.732	0.742	0.055	0.72	0.779	0.115	0.27	0	0.359	0.350	0.029	0.343	0.348	0.020	0.08	0
**Posterior cingulate**	0.774	0.777	0.056	0.81	0.810	0.047	0.01	1	0.333	0.343	0.042	0.306	0.307	0.039	0.01	1
**Supplementary motor cortex**	0.708	0.697	0.041	0.68	0.686	0.095	0.38	0	0.385	0.393	0.028	0.374	0.378	0.022	0.02	1
**Central operculum**	0.69	0.71	0.11	0.681	0.690	0.074	0.45	0	0.361	0.376	0.041	0.373	0.376	0.021	0.71	0
**Triangular part of inferior frontal gyrus**	0.749	0.751	0.071	0.73	0.732	0.068	0.37	0	0.343	0.346	0.045	0.353	0.354	0.033	0.18	0
**Middle frontal gyrus**	0.734	0.738	0.052	0.71	0.736	0.069	0.27	0	0.367	0.365	0.029	0.377	0.378	0.022	0.04	1
**Superior frontal gyrus and frontal pole**	0.722	0.714	0.073	0.67	0.689	0.107	0.02	1	0.362	0.362	0.043	0.367	0.375	0.027	0.86	0
**Precentral gyrus**	0.690	0.702	0.072	0.74	0.772	0.131	4 $\times \;10^{-3}$	1	0.389	0.392	0.027	0.333	0.354	0.098	3 $\times \;10^{-5}$	1
**Superior temporal gyrus**	0.689	0.673	0.059	0.65	0.663	0.100	0.16	0	0.377	0.383	0.022	0.359	0.363	0.019	2 $\times \;10^{-4}$	1
**Middle temporal gyrus**	0.578	0.556	0.088	0.67	0.682	0.088	3 $\times \;10^{-5}$	1	0.376	0.378	0.016	0.372	0.372	0.015	0.23	0
**Right Hemisphere**																
	**Shannon Entropy**								**Statistical Complexity**							
**Region**	**Female**			**Male**			**Test**		**Female**			**Male**			**Test**	
	**mean**	**median**	**sd**	**mean**	**median**	**sd**	***p*-value**	**h**	**mean**	**median**	**sd**	**mean**	**median**	**sd**	***p*-value**	**h**
**Postcentral gyrus (including medial segment)**	0.625	0.640	0.091	0.697	0.701	0.084	1 $\times \;10^{-3}$	1	0.378	0.381	0.026	0.363	0.369	0.025	0.04	1
**Middle frontal gyrus**	0.765	0.778	0.071	0.795	0.802	0.056	0.01	1	0.349	0.355	0.044	0.326	0.320	0.039	5 $\times \;10^{-3}$	1
**Superior temporal gyrus**	0.731	0.734	0.071	0.650	0.657	0.082	1 $\times \;10^{-6}$	1	0.359	0.366	0.030	0.370	0.369	0.014	0.2	0
**Middle temporal gyrus**	0.71	0.79	0.12	0.73	0.77	0.10	0.9	0	0.331	0.313	0.036	0.341	0.339	0.023	0.1	0

**Table 6:** Results of the Benjamini–Hochberg FDR correction and significance analysis. Light gray shading indicates cases where sex differences were identified.

**Left Hemisphere**												
	**D = 6**				**D = 6 (5 Patients)**				**D = 5**			
	**H**		**C**		**H**		**C**		**H**		**C**	
**Region**	$pv_{c}$	**Ho**	$pv_{c}$	**Ho**	$pv_{c}$	**Ho**	$pv_{c}$	**Ho**	$pv_{c}$	**Ho**	$pv_{c}$	**Ho**
**Superior parietal lobule**	0.11	F	2.10 $\times \;10^{-4}$	T	0.09	F	2.10 $\times \;10^{-4}$	T	0.17	F	1.18 $\times \;10^{-4}$	T
**Supramarginal gyrus**	2.27 $\times \;10^{-5}$	T	0.13	F	2.40 $\times \;10^{-5}$	T	0.14	F	1.62 $\times \;10^{-5}$	T	0.14	F
**Precuneus**	0.36	F	0.13	F	0.36	F	0.14	F	0.16	F	0.14	F
**Posterior cingulate**	0.03	T	0.04	T	0.02	T	0.03	T	0.04	T	0.03	T
**Supplementary motor cortex**	0.41	F	0.06	F	0.43	F	0.05	F	0.13	F	0.05	F
**Central operculum**	0.45	F	0.76	F	0.48	F	0.76	F	0.85	F	0.76	F
**Triangular part of inferior frontal gyrus**	0.41	F	0.22	F	0.43	F	0.24	F	0.24	F	0.24	F
**Middle frontal gyrus**	0.36	F	0.11	F	0.36	F	0.08	F	0.99	F	0.09	F
**Superior frontal gyrus and frontal pole**	0.05	F	0.86	F	0.04	T	0.86	F	0.64	F	0.86	F
**Precentral gyrus**	0.01	T	2.10 $\times \;10^{-4}$	T	0.01	T	2.10 $\times \;10^{-4}$	T	0.99	F	1.40 $\times \;10^{-4}$	T
**Superior temporal gyrus**	0.26	F	9.33 $\times \;10^{-4}$	T	0.26	F	9.33 $\times \;10^{-4}$	T	0.63	F	7.00 $\times \;10^{-4}$	T
**Middle temporal gyrus**	1.58 $\times \;10^{-4}$	T	0.26	F	1.60 $\times \;10^{-4}$	T	0.26	F	0.02	T	0.28	F
**Right Hemisphere**												
	**D = 6**				**D = 6 (5 patients)**				**D = 5**			
	**H**		**C**		**H**		**C**		**H**		**C**	
**Region**	$pv_{c}$	**Ho**	$pv_{c}$	**Ho**	$pv_{c}$	**Ho**	$pv_{c}$	**Ho**	$pv_{c}$	**Ho**	$pv_{c}$	**Ho**
**Postcentral gyrus (including medial segment)**	0.36	F	0.13	F	4.00 $\times \;10^{-3}$	T	0.08	F	0.44	F	0.34	F
**Middle frontal gyrus**	0.18	F	0.13	F	0.02	T	0.02	T	0.17	F	0.15	F
**Superior temporal gyrus**	5.18 $\times \;10^{-6}$	T	0.13	F	0.00	T	0.25	F	6.88 $\times \;10^{-6}$	T	6.88 $\times \;10^{-6}$	T
**Middle temporal gyrus**	0.12	F	0.16	F	0.90	F	0.15	F	0.13	F	0.10	F

## Data Availability

The dataset used in this study was sourced from the Montreal Neurological Institute (MNI) Open iEEG Atlas, which is publicly accessible at https://mni-open-ieegatlas.research.mcgill.ca/, accessed on June 2024. This resource is a collaborative effort involving the Montreal Neurological Institute and Hospital, the Grenoble-Alpes University Hospital, and the Centre hospitalier de l’Université de Montréal. The Atlas provides a comprehensive collection of normal intracranial EEG data. For more information about this dataset, refer to the references listed in the bibliography [49,50,51].
